# Fc-mediated antibody functions are associated with disease severity in COVID-19

**DOI:** 10.3389/fimmu.2026.1797975

**Published:** 2026-05-01

**Authors:** Sungim Choi, Jong Su Kang, Jaehwan You, Man-Seong Park, Hee Bum Jo, Ji-Soo Kwon, Sung-Han Kim, Seong Yeon Park

**Affiliations:** 1 Division of Infectious Diseases, Department of Internal Medicine, Dongguk University Ilsan Hospital, Goyang-si, Gyeonggi-do, Republic of Korea; 2 Department of Microbiology, Institute for Viral Diseases, College of Medicine, Korea University, Seoul, Republic of Korea; 3 Department of Microbiology, Vaccine Innovation Center, Institute for Viral Diseases, College of Medicine, Korea University, Seoul, Republic of Korea; 4 Division of Infectious Diseases, Department of Internal Medicine, Incheon Sejong Hospital, Incheon, Republic of Korea; 5 Department of Infectious Diseases, Asan Medical Center, University of Ulsan College of Medicine, Seoul, Republic of Korea

**Keywords:** antibody-dependent cellular phagocytosis, antibody-dependent enhancement, Fc-mediated functions, inflammation, SARS-CoV-2

## Abstract

**Introduction:**

Fc-mediated antibody effector functions play key roles in both antiviral immunity and immunopathology in COVID-19. While antibody-dependent cellular phagocytosis (ADCP) and antibody-dependent cellular cytotoxicity (ADCC) contribute to pathogen clearance, they may also be associated with inflammatory responses. The contribution of these pathways—particularly antibody-dependent enhancement (ADE)—to severe COVID-19 remains incompletely understood. We prospectively investigated relationships among ADE, ADCC, and ADCP across multiple SARS-CoV-2 variants from 64 patients with laboratory-confirmed SARS-CoV-2 infection.

**Methods:**

Patient Fc-mediated effector activities were profiled. Disease severity was classified using National Institutes of Health criteria. ADE was assessed using pseudotyped SARS-CoV-2 entry into THP-1 cells, whereas Fc gamma receptors (FcγR)IIIa- and FcγRIIa-mediated reporter activities (reflecting ADCC and ADCP surrogate responses, respectively) were assessed using spike-expressing A549 target cells and FcγR-expressing Jurkat-Lucia reporter cells. Functional responses were measured against wild-type SARS-CoV-2 and Omicron sublineages (B.1.1.529, BA.4/BA.5, XBB.1.5).

**Results:**

Among 64 participants (40 with mild, 24 with severe COVID-19; median age, 58 years [interquartile range, 45–71], 56% men), anti-spike IgG binding did not differ by severity. ADE activity was significantly higher in severe than mild COVID-19 and remained independently associated with severity in multivariable analysis. FcγRIIa-mediated reporter activity was higher in severe cases in descriptive and univariate analyses, but lost statistical significance after adjustment for age, sex, comorbidities, and variant period. FcγRIIIa-mediated reporter activity did not differ by severity but was reduced against Omicron variants. Plasma from severe cases promoted enhanced FcγR-dependent viral entry in pseudovirus assays; yet, subsequent live Omicron virus infection did not lead to an increase in viral RNA replication—consistent with an abortive infection without evidence of productive viral replication.

**Conclusion:**

Our findings suggest that ADE activity is associated with dysregulated Fc-mediated immune responses in severe COVID-19. Although multiple Fcγ receptor–mediated pathways were observed, the independent association of ADE with disease severity supports a potential link with excessive FcγR-mediated myeloid activation. FcγRIIa reporter activity likely reflects overlapping immune signatures rather than an independent pathogenic contribution, whereas FcγRIIIa reporter activity showed no clear relationship with clinical outcomes. These findings highlight the complexity of Fc-effector immunity and support further investigation into therapeutic strategies targeting Fc–FcγR interactions.

## 1 Introduction

The development and widespread deployment of vaccines have substantially reduced the global burden of coronavirus disease 2019 (COVID-19). However, the continual emergence of novel SARS-CoV-2 variants—particularly those with extensive mutations in the spike protein—has sustained transmission and periodically undermined vaccine effectiveness, posing persistent public health challenges (1). While most infected individuals experience asymptomatic or mild disease, a substantial proportion progresses to severe illness, requiring hospitalization or intensive care (2, 3). This clinical heterogeneity underscores the need to elucidate the immunopathological mechanisms underlying severe COVID-19 outcomes.

Severe manifestations of COVID-19 are often accompanied by hyperinflammatory responses, dysregulated immunity, and aberrant complement activation, all of which are associated with adverse clinical trajectories (4–6). In vaccinated individuals, higher SARS-CoV-2-specific neutralizing antibody (nAb) titers correlate with protection against symptomatic infection (7). However, elevated titers of nAbs have been paradoxically linked to greater disease severity, suggesting that humoral immune responses may contribute not only to viral control but also to immunopathology (8, 9). Moreover, SARS-CoV-2 variants have acquired numerous receptor-binding domain mutations, rendering them more immune-evasive and diminishing the efficacy of previously effective neutralizing antibodies (10, 11). This highlights the need to explore antibody functions beyond neutralization, particularly Fc-mediated effector mechanisms.

Immunoglobulin G (IgG) antibodies possess an Fc domain capable of engaging innate immune cells through Fc gamma receptors (FcγRs), thereby mediating functions such as antibody-dependent cellular cytotoxicity (ADCC) and antibody-dependent cellular phagocytosis (ADCP). These effector pathways enhance viral clearance by activating macrophages, natural killer (NK) cells, and neutrophils. However, when dysregulated, aberrant FcγR engagement may also exacerbate tissue damage and inflammation. Of particular concern is antibody-dependent enhancement (ADE), a phenomenon in which non-neutralizing or sub-neutralizing antibodies facilitate viral entry into FcγR-expressing cells—such as monocytes and macrophages—potentially modulating the inflammatory landscape and immune-mediated pathology (12, 13). Notably, the functional potential of these Fc-mediated pathways can be characterized using FcγR-mediated reporter systems, which serve as established surrogates for measuring the initiation of effector activities.

Importantly, Fc-effector profiles vary across individuals, disease severities, and viral variants; yet, the clinical implications of these differences remain incompletely understood. While ADCC and ADCP are generally regarded as beneficial in antiviral defense, their precise role in COVID-19 is complex. For instance, some studies have shown that robust ADCC and ADCP activities correlate with milder disease course and survival (14, 15), whereas others have observed elevated ADCC levels in critically ill patients, suggesting a potential role in driving hyperinflammation through excessive pro-inflammatory cytokine release (16, 17). Furthermore, although ADE is well-documented in viruses like Dengue, its presence in COVID-19 remains intensely debated. While *in vitro* studies have occasionally reported low-level ADE using specific monoclonal antibodies or convalescent sera (18), large-scale clinical evidence has mostly failed to confirm ADE as a primary driver of severe COVID-19 pathology, contrasting with its role in other respiratory infections (19, 20).

Therefore, this study investigated the relationship between Fc-mediated antibody functions and COVID-19 severity by analyzing plasma samples from patients with varying clinical presentations. We assessed ADE activity and measured FcγRIIIa- and FcγRIIa-mediated reporter activities as surrogates for ADCC and ADCP, respectively, These functions were evaluated against multiple SARS-CoV-2 variants, including the ancestral Wuhan-hu-1 strain, Delta, and Omicron sublineages (B.1.1.529, BA.4/BA.5, and XBB.1.5). By analyzing the Fc-functional antibody profiles in both mild and severe cases, we aimed to clarify how these effector potentials relate to clinical outcomes. Our findings may contribute to a better understanding of the balance between protective and potentially inflammatory Fc-mediated responses, which remains relevant for the refined design of future antibody-based interventions.

## 2 Materials and methods

### 2.1 Human samples

Peripheral blood samples were prospectively collected from patients with SARS-CoV-2 infection between January 2021 and January 2024 from two institutions in South Korea: Dongguk University Ilsan Medical Center (a secondary care hospital) and Asan Medical Center (a tertiary referral center). SARS-CoV-2 infection was confirmed using reverse transcriptase–polymerase chain reaction testing of nasopharyngeal swab specimens. Clinical and demographic data, including age, sex, comorbidities, and laboratory parameters, were obtained from electronic medical records.

Eligible participants were enrolled within 1 month of symptom onset and stratified into mild and severe disease groups according to the National Institutes of Health (NIH) COVID-19 severity classification criteria (21). Patients categorized as mild exhibited no or minimal respiratory symptoms without evidence of pneumonia or hypoxia (NIH severity score ≤ 3), whereas severe cases (NIH severity score 4–5) included individuals requiring oxygen supplementation or mechanical ventilation.

Although the first Omicron variant cases in South Korea were identified in early December 2021, the variant became dominant in January 2022, accounting for over 50% of sequenced cases by mid-January (22, 23). Accordingly, to assess the impact of viral variants on immune responses, patients were further categorized into two groups based on their diagnosis date: “pre-Omicron” (diagnosed on or before December 31, 2021) and “post-Omicron” (diagnosed on or after January 1, 2022).

All study protocols were reviewed and approved by the Institutional Review Boards of Dongguk University Ilsan Medical Center (IRB No. 2023-08-013) and Asan Medical Center (IRB No. 2023-0883). Written informed consent was obtained from all participants or their legal guardians in accordance with the principles of the Declaration of Helsinki.

### 2.2 Cell culture, chemicals, and proteins

THP-1(TIB-202), 293T (CRL-3216), and A549 (CRM-CCL-185) cells were purchased from ATCC (Manassas, VA, USA). Jurkat-Lucia™ NFAT-CD32 and Jurkat-Lucia™ NFAT-CD16 cells were purchased from InvivoGen (CA, USA). Dulbelco’s modified media (DMEM), RPMI-1640, Phosphate-buffered saline (PBS), and penicillin/streptomycin (Pen/Strep) were purchased from WELGENE (Daegu, Korea). Iscove’s Modified Dulbecco’s Medium (IMDM) was purchased from InvivoGen (CA, USA). 293T cells and A549 cells were cultured in DMEM containing 10% fetal bovine serum (FBS) and 1% penicillin/streptomycin (Pen/Strep) at 37 °C with 5% CO_2_. THP-1 cells were cultured in RPMI-1640 containing 0.05 mM β-merchaptoethanol (Sigma-Aldrich, MO, USA) and 10% FBS at 37 °C with 5% CO_2._ Jurkat-Lucia™ NFAT-CD32 and Jurkat-Lucia™ NFAT-CD16 cells were cultured in IMDM containing 10% FBS, 100 µg/mL Normocin™, and 1% penicillin/streptomycin (Pen/Strep) at 37 °C with 5% CO_2_. Lipofectamine™ 3000 transfection reagent was purchased from Thermo Fisher Scientific (Waltham, MA, USA). SARS-CoV-2 (2019-nCoV) Spike S1 (45951-V08H), SARS-CoV-2 (B.1.1.529 sublineage BA.2) Spike S1 (40591-V08H43), SARS-CoV-2 (BA.4/BA.5/BA.5.2) Spike S1(40591-V08H46), and SARS-CoV-2 (XBB.1.5) Spike S1 (40591-V08H47) proteins were purchased from SinoBiological (Beijing, China).

### 2.3 Generation of A549 cells expressing SARS-CoV-2 spike variants

pLENTI-hACE2-puro (#155295), pCMV14-3x-Flag-SARS-CoV-2-S(#145780), pcDNA3.3_SARS2_omicron_BA.1 (#180375), pcDNA3.3_SARS2_omicron_BA.1 (#183700), pMD2.G (#12259), and psPAX2 (#12260) plasmids were purchased from Addgene (Cambridge, MA, USA). pLV-8 plasmid was acquired from Invivogen (San Diego, CA, USA).

Lentiviral expression plasmids (pLENTI-SARS-CoV-2-S-puro, pLENTI-SARS-CoV-2-spike delta S-puro, pLENTI-SARS-CoV-2-omicron_BA.1-puro, and pLENTI-SARS-CoV-2-omicron_BA.2-puro) were generated by replacing the hACE2 coding sequence in the pLENTI-hACE2-puro backbone with sequences encoding SARS-CoV-2 spike variants. 293T cells were transfected with 4.3 µg lentiviral expression plasmid together with lentiviral helper plasmids (9 µg psPAX2 and 4 µg pMD2.G) for 72 h. Viral supernatant was then collected, filtered, and transduced into A549 cells, which were selected with puromycin (3 µg/mL) for 48 h.

### 2.4 Generation of pseudotyped-SARS-CoV-2 virus

293T cells (5.0 × 10^6^) were seeded in a 100-mm culture dish and incubated overnight. Subsequently, the cells were transfected with a combination of the following plasmids: pHAGE-CMV-Luc2-IRES-ZsGreen-W (#164432, Addgene) 4.3 µg, psPAX2 (#12260, Addgene) 9 µg, and pCMV14-3x-Flag-SARS-CoV-2 S (#145780, Addgene) 4 µg.

Lipofectamine™ 3000 reagent was used for transfection following the manufacturer’s protocols. At 6 h post-transfection, the plate medium was changed with DMEM containing 10% FBS. Twenty-four h after transfection, the supernatant was harvested, replaced with DMEM containing 10% FBS, and stored at 4 °C. Fifty-two h after transfection, the supernatant was harvested and combined with the 24-h supernatant. To remove cellular debris, the supernatant mixtures were centrifuged at 2,000 rpm for 10 min and filtered using a 0.45-µm filter. The filtered mixtures were aliquoted and stored at -80 °C.

### 2.5 SARS-CoV-2 S1 spike–IgG enzyme-linked immunosorbent assay assay

Recombinant S1 antigens of SARS-CoV-2 (derived from the wild-type strain and major variants), including 2019-nCoV, B.1.1.529 sublineage BA.2, BA.4/BA.5/BA.5.2, and XBB.1.5, at 2 µg/mL, were coated onto 96-well immuno plates (Thermo Fisher Scientific, Waltham, MA, USA) overnight at 4 °C. The plates were subsequently incubated with blocking solution (1x PBS containing 1% bovine serum albumin) for 1 h at room temperature. After washing three times with 1×PBST, diluted (1:20,000) plasma was added and incubated for 2 h at room temperature. After washing five times with 1×PBST, horseradish peroxidase-conjugated anti-human IgG solution (1:20.000, Jackson Immunoresearch, West Grove, PA) was incubated for 1 h at room temperature. After washing seven times with 1×PBST, plates were developed with 3, 3’, 5, 5’-tetramethylbenzidine substrates (Sigma-Aldrich, MO, USA) for 30 min, and the reaction was stopped with a stop solution (Sigma-Aldrich, MO, USA). Optical density (OD)—serving as a surrogate marker of antigen-specific IgG binding activity—was obtained using a SPARK multimode reader (TECAN, Switzerland) and was measured at 450 nm. To determine the cut-off values for the ELISA, we measured the mean values and standard deviations (SDs) of the OD values of 10 negative control, SARS-CoV-2-naïve, samples. The cut-off values were determined by calculating the mean OD plus three times the SD values.

### 2.6 ADE assay

#### 2.6.1 Pseudotyped SARS-CoV-2 assay

Plasma samples were incubated for 30 min at 56 °C and then serially diluted 10-fold in 1xPBS (dilution range: 1:4 to 1:400000). In a 96-well plate, pseudotyped SARS-CoV-2 virus particles (5.0×10^4^ TCID_50_/mL) were incubated with serially diluted serum for 1 h at 37 °C with 5% CO_2_. THP-1 cells (2×10^6^ cells/mL) were added to serum combined with the pseudovirus mixture and incubated for 24 h. Cell infection was evaluated based on luciferase expression using the Bright-Glo™ Luciferase Assay System (Promega, Madison, WI, USA). Luminescence was measured using a SPARK multimode reader (TECAN, Switzerland). Plasma samples collected before the COVID-19 pandemic (2019) were used as negative controls to establish baseline ADE activity in the pseudotyped SARS-CoV-2 assay.

#### 2.6.2 Live SARS-CoV-2 (Omicron) virus assay

Serial 10-fold dilutions of plasma or antibody samples (25 µL per well) were prepared and added to each well of a 96-well plate. Subsequently, 25 µL of SARS-CoV-2 Omicron BA.5 virus (hCoV-19/Korea/KDCA17739542, lineage BA.5, NCCP43426) with a multiplicity of infection of 0.5 was added to each well. The plasma–virus mixtures were incubated for 1 h at 37 °C in a humidified incubator containing 5% CO_2_. After incubation, the mixtures were added to Fcγ receptor-positive THP-1 cells (1×10^5^ cells) seeded in 96-well plates and further incubated for 24 h. Subsequently, cell pellets and supernatants were collected for viral RNA quantification.

Viral replication was quantified using RT-qPCR targeting the SARS-CoV-2 RNA-dependent RNA polymerase (RdRp) gene (forward primer: 5’-GTGAAATGGTCATGTGTGGCGG-3’; reverse primer: 5’-CAAATGTTAAAAACACTATTAGCATA-3’), as described in the Charité protocol (24). The RdRp RNA levels were normalized to GAPDH expression (forward primer: 5’-ACCACAGTCCATGCCATCAC-3’; reverse primer: 5’-TCCACCACCCTGTTGCTGTA-3’).

Plasma samples collected before the COVID-19 pandemic (2019) were used as negative controls. A recombinant SARS-CoV-2 spike monoclonal antibody with reported ADE activity (clone, S9HC; catalog no. A02133; GenScript) was included as a positive control to confirm that the assay system can detect Fcγ receptor-mediated viral entry enhancement. The 24 h post-infection time point was selected to capture early Fcγ receptor-mediated viral uptake rather than productive viral replication, as SARS-CoV-2 infection in monocytes/macrophages is often abortive. The same time frame was applied across Fc effector function assays to ensure consistency with ADCC and ADCP assays. All experiments involving infectious SARS-CoV-2 were performed in a certified biosafety level 3 (BSL-3) facility at Korea University, supported by the K-Vaccine Innovation Center (grant no. 2021R1A6C101C570), Korea University College of Medicine.

#### 2.6.3 Area under the dilution–response curve calculation

For a quantitative comparison of ADE among individuals, the AUC was calculated for each patient using the trapezoidal integration method, based on the log_10_ of serum dilution plotted on the x-axis and the relative luciferase signal on the y-axis. AUC was selected as a robust, continuous variable representing the overall magnitude of enhancement across all dilutions, which minimizes variability from single-point measurements.

### 2.7 FcγRIIIa mediated reporter assay (ADCC surrogate) activity

To evaluate ADCC potential across SARS-CoV-2 variants, we measured FcγRIIIa-mediated reporter activity using spike-expressing A549 target cells and Jurkat-Lucia™ NFAT-CD16 effector cells. Instead of using recombinant spike proteins, stable A549 cell lines expressing SARS-CoV-2 spike variants were employed as target cells to better mimic cell-surface antigen presentation. Jurkat-Lucia™ NFAT reporter cells, which stably express a Lucia luciferase gene under the control of an ISG54 minimal promoter fused to NFAT response elements, were used to quantify FcγRIIIa activation.

Plasma samples were heat-inactivated at 56 °C for 30 min and diluted 1:100 in culture medium. Spike-expressing A549 cells (5.0 × 10^5^ cells/mL) were seeded in 96-well plates and incubated for 24 h at 37 °C. After washing three times with 1×PBST, diluted plasma was added and incubated for 24 h at 37 °C, followed by the addition of Jurkat-Lucia™ NFAT-CD16 cells (1.0 × 10^6^ cells/mL) for a further 6 h incubation at 37 °C. Luciferase activity was measured using QUANTI-Luc™ (InvivoGen, CA, USA), and luminescence was detected using a SPARK multimode reader (TECAN, Switzerland).

The use of PBST washing and extended plasma incubation was applied to support immune complex formation and consistent reporter signal detection across samples.

### 2.8 FcγRIIa mediated reporter assay (ADCP surrogate) activity

To evaluate FcγRIIa-mediated reporter activity as a surrogate of ADCP, we used spike-expressing A549 cells as target cells and Jurkat-Lucia™ NFAT-CD32 cells as effector cells. These effector cells stably express the human FcγRIIa (CD32) and a Lucia luciferase reporter gene under the control of an ISG54 minimal promoter fused to NFAT response elements.

The assay was performed under conditions similar to those described for the FcγRIIIa-mediated reporter assay.

Briefly, spike-expressing A549 cells were seeded and incubated for 24 h. After washing three times with 1×PBST, diluted plasma added and incubated for 24h at 37 °C to facilitate immune complex formation. Subsequently, Jurkat-Lucia™ NFAT-CD32 cells (1.0 × 10^6^ cells/mL) were added and incubated for 6 h at 37 °C. Reporter activity was quantified by measuring luciferase activity using QUANTI-Luc™ (InvivoGen, CA, USA), and luminescence was detected using a SPARK multimode reader (TECAN, Switzerland).

The use of PBST washing and extended plasma incubation was applied to support immune complex formation and consistent reporter signal detection across samples.

### 2.9 Association of ADE activity with systemic inflammatory markers and circulating cytokines

To further explore the relationship between ADE activity and systemic inflammation, we analyzed inflammatory markers and circulating cytokines from plasma samples collected from patients with COVID-19.

C-reactive protein (CRP) levels were obtained from clinical laboratory data, and the neutrophil-to-lymphocyte ratio (NLR) was calculated from complete blood count parameters. Interleukin (IL)-6 and tumor necrosis factor (TNF)-α levels were quantified using commercial ELISA kits (Koma Biotech, K0331194; R&D Systems, DTA00D) according to the manufacturer’s instructions. For IL-6 measurement, diluted plasma samples (1:3) were added in duplicate to pre-coated 96-well plates and incubated for 2 h at room temperature. Plates were washed four times with 1× washing buffer, followed by incubation with diluted detection antibody for 2 h at room temperature. After washing, diluted streptavidin–HRP solution was added and incubated for 30 min at room temperature. Plates were then washed and developed with TMB substrate for 20 min, and the reaction was stopped by adding a stop solution. OD was measured at 450 nm using a SPARK multimode reader (Tecan, Switzerland). For TNF-α measurement, diluted plasma samples (1:3) were added in duplicate to pre-coated 96-well plates and incubated for 2 h at room temperature. The plates were washed four times with 1× washing buffer, followed by incubation with human TNF-α conjugate solution for 2 h at room temperature. After washing, the plates were developed with TMB substrate for 30 min, and the reaction was stopped by adding a stop solution. OD was measured at 450 nm using a SPARK multimode reader (Tecan, Switzerland).

### 2.10 Statistical analysis

Continuous variables were summarized as medians with interquartile ranges (IQRs), and categorical variables as counts and percentages. Baseline differences between mild and severe COVID-19 groups were compared using the Mann–Whitney U test for continuous variables and Fisher’s exact test for categorical variables. Due to their skewed distributions, Fc-mediated reporter activities (surrogates for ADCC and ADCP), ADE activity, and inflammatory markers were primarily compared between groups using non-parametric Mann–Whitney U tests. For analyses involving normally distributed data or repeated measures across variants, Welch’s t-test and one-way repeated measures analysis of variance were applied as appropriate.

Both univariate and multivariable logistic regression analyses were conducted. To address the right-skewed distribution and reduce the influence of extreme values inherent to Fc-mediated antibody functions measurements, ADE, FcγRIIIa-, and FcγRIIa-mediated reporter activities were log-transformed before regression analyses. Univariate analyses evaluated the crude association between each Fc-mediated antibody function and disease severity without adjustment. Multivariate models included age, sex, presence of any comorbidity, and the SARS-CoV-2 variant period (pre-Omicron vs post Omicron) as covariates. To prevent overfitting given the modest number of severe cases, covariate selection was strictly limited in accordance with events-per-variable recommendations for logistic regression. Odds ratios (ORs) and 95% confidence intervals (CIs) were calculated by exponentiating model coefficients.

Correlations between ADE activity (AUC) and inflammatory markers were evaluated using Spearman’s rank correlation coefficient. All analyses were performed using GraphPad Prism 10.1.2 (San Diego, CA, USA) and R version 4.3.2 (R Foundation for Statistical Computing, Vienna, Austria). All statistical tests were two-sided, and *p*-values < 0.05 were considered statistically significant.

## 3 Results

### 3.1 Clinical characteristics of patients with mild or severe COVID-19

A total of 64 patients were enrolled, including 40 with mild and 24 with severe disease (NIH severity criteria). Baseline patient characteristics are summarized in **Table 1**. The severe disease group included significantly older individuals than the mild disease group (mean age: 73.0 vs. 54.1 years, *p* < 0.01). Patients with severe disease also had a markedly higher prevalence of comorbidities (95.8% vs. 35.0%, *p* < 0.001), particularly hypertension (83.3% vs. 32.5%, *p* < 0.01).

**Table 1:** Baseline characteristics in patients with mild and severe coronavirus disease 2019 (COVID-19).

Characteristic	Mild (n = 40)	Severe (n = 24)	*p*-value
Age in years, mean (SD)	54.1± 18.8	73.0 ± 9.4	< 0.01
Male, n (%)	14 (35.0%)	14 (58.3%)	0.14
Symptoms at presentation, n (%)			
Fever	19 (47.5%)	10 (41.7%)	0.24
Chill	5 (12.5%)	3 (12.5%)	> 0.99
Cough	16 (40.0%)	12 (50.0%)	> 0.99
Sputum	12 (30.0%)	10 (41.7%)	> 0.99
Sore throat	10 (25.0%)	2 (8.3%)	0.05
Dyspnea	4 (10.0%)	14 (58.3%)	< 0.01
Rhinorrhea	3 (7.5%)	0 (0.0%)	0.25
Hemoptysis	0 (0.0%)	0 (0.0%)	> 0.99
Chest pain	1 (2.5%)	0 (0.0%)	> 0.99
Diarrhea	4 (10.0%)	1 (4.2%)	0.37
Headache	8 (20.0%)	2 (8.3%)	0.16
Myalgia	9 (22.5%)	3 (12.5%)	0.19
Nasal congestion	1 (2.5%)	0 (0.0%)	> 0.99
Hyposmia	0 (0.0%)	1 (4.2%)	0.44
Hypogeusia	1 (2.5%)	2 (8.3%)	0.57
Pneumonia	1 (2.5%)	19 (79.2%)	< 0.01
Comorbidities, n (%)			
Any comorbidities	14 (35.0%)	23 (95.8%)	< 0.01
Diabetes	6 (15.0%)	11 (45.8%)	0.06
Chronic lung diseases	1 (2.5%)	0 (0.0%)	> 0.99
Hypertension	13 (32.5%)	20 (83.3%)	< 0.01
Cardiovascular diseases	4 (10.0%)	2 (8.3%)	0.69
Chronic kidney diseases	1 (2.5%)	3 (12.5%)	0.30
Chronic liver diseases	0 (0.0%)	1 (4.2%)	0.43
Solid cancer	2 (5.0%)	3 (12.5%)	0.64
Hematologic cancer	3 (7.5%)	4 (16.7%)	0.45
Transplantation	0 (0.0%)	3 (12.5%)	0.07
HIV infection	0 (0.0%)	1 (4.2%)	0.43
Outcomes, n (%)			
ICU admission	2 (5.0%)	11 (45.8%)	< 0.01
Ventilator use	2 (5.0%)	9 (37.5%)	0.01
In-hospital mortality	0 (0.0%)	6 (25.0%)	< 0.01
Initial laboratory data, mean (SD)			
WBC (/uL)	6903.3 ± 2978.0	10304.6 ± 7569.0	0.06
Neutrophil (%)	60.7 ± 17.7	80.9 ± 11.5	< 0.01
Lymphocyte (%)	29.3 ± 16.8	11.4 ± 7.3	< 0.01
Hgb (g/dL)	12.0 ± 2.1	11.9 ± 2.9	0.94
Platelets (10^3^/uL)	220.4 ± 82.1	194.5 ± 101.2	0.32
BUN (mg/dL)	12.9 ± 8.3	28.0 ± 25.3	< 0.01
Creatinine (mg/dL)	0.8 ± 0.4	1.4 ± 1.9	0.37
AST (IU/L)	35.8 ± 40.6	59.7 ± 38.0	< 0.01
ALT (IU/L)	29.1 ± 38.3	40.4 ± 27.4	0.01
C-reactive protein (mg/dL)	3.0 ± 4.7	9.2 ± 9.3	< 0.01

Values are expressed as n (%), or mean ± SD, as appropriate. *P*-values were calculated using the Mann–Whitney U test or Fisher’s exact test, as appropriate.

SD, standard deviation; n, number; HIV, human immunodeficiency virus; ICU, intensive care unit; WBC, white blood cell count; Hgb, hemoglobin; BUN, blood urea nitrogen; AST, aspartate aminotransferase; ALT, alanine aminotransferase.

Clinically, patients with severe disease were more likely to have dyspnea (58.3% vs. 10.0%, *p* < 0.01) and radiologic evidence of pneumonia (79.2% vs. 2.5%, *p* < 0.01). Outcomes such as ICU admission (45.8% vs. 5.0%, *p* < 0.01), ventilator use (37.5% vs. 5.0%, *p* = 0.01), and in-hospital mortality (25.0% vs. 0.0%, *p* < 0.01) were significantly worse in the severe disease group. Laboratory data on admission showed elevated inflammatory markers in severe cases, including higher neutrophil percentages (80.9% vs. 60.7%, *p* < 0.01), lower lymphocyte percentages (11.4% vs. 29.3%, *p* < 0.01), increased C-reactive protein (6.92 vs. 0.69 mg/dL, *p* < 0.01), and elevated aspartate aminotransferase and alanine aminotransferase levels (*p* < 0.05), consistent with systemic inflammation.

### 3.2 Anti-SARS-CoV-2 S1 IgG responses

**Figure 1:**
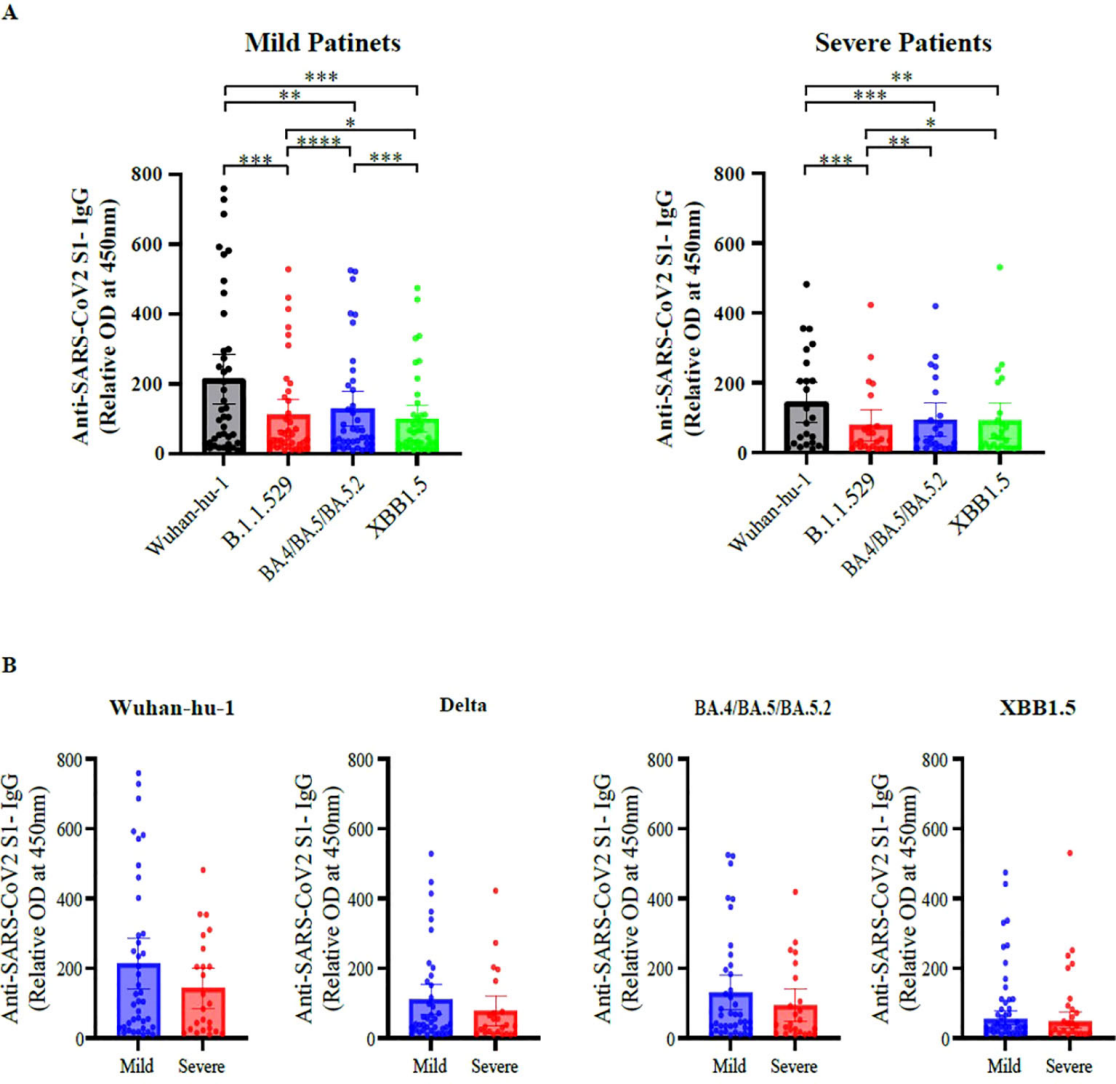
Plasma immunoglobulin (Ig)G binding to SARS-CoV-2 spike S1 across variants. **(A)** Plasma IgG binding to the spike subunit 1 (S1) protein of SARS-CoV-2 variants (Wuhan-Hu-1, B.1.1.529, BA.4/BA.5/BA.5.2, and XBB.1.5) was measured by ELISA in patients with mild and severe COVID-19. Data are presented as relative optical density (OD) at 450 nm. Comparisons among variants within each severity group were performed using one-way repeated measures analysis of variance. **(B)** Comparison of plasma IgG binding between patients with mild and severe COVID-19 for each SARS-CoV-2 variant Each dot represents an individual patient; bars indicate mean ± standard error of the mean. Differences between two groups were analyzed using the Mann–Whitney U test. Sample sizes were as follows: mild (n = 40) and severe (n = 24) patients. All tests were two-sided, and *p*-values < 0.05 were considered statistically significant. *p < 0.05, **p < 0.01, ***p < 0.001, ****p < 0.0001 COVID-19, coronavirus disease 2019; ELISA, enzyme-linked immunosorbent assay.

We performed ELISAs to quantify plasma IgG binding to the SARS-CoV-2 spike subunit 1 (S1) protein. As shown in **Figure 1A**, both patient groups (mild and severe disease) exhibited markedly reduced OD values for all tested variants than for the wild-type strain, indicating attenuated antibody binding, likely attributable to spike protein mutations. However, no significant differences in OD values were observed between the mild and severe disease groups for any individual variant (**Figure 1B**). These results suggest that, although antigenic drift in emerging variants compromises antibody binding efficiency, the magnitude of IgG recognition did not differ according to disease severity in this cohort.

### 3.3 Antibody-dependent enhancement

**Figure 2:**
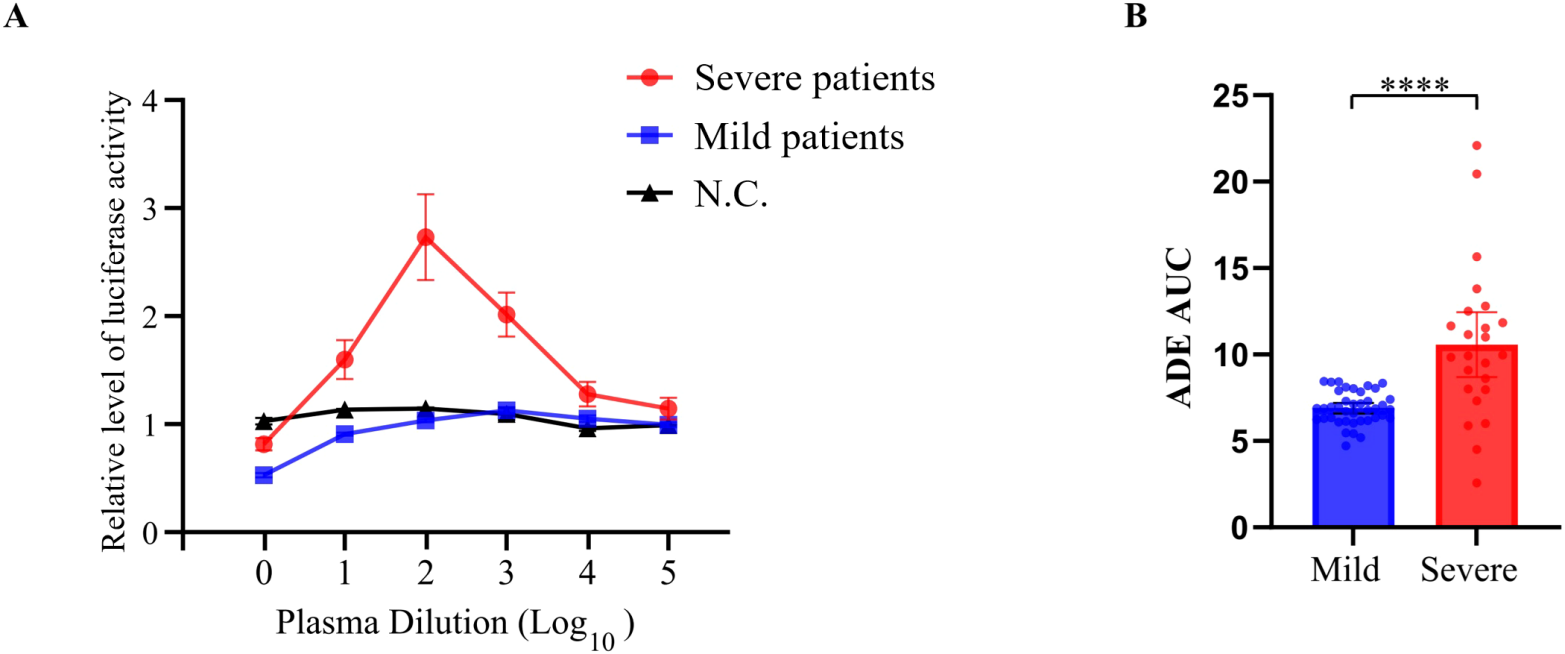
Antibody-dependent enhancement (ADE) activity in patients with coronavirus disease 2019 (COVID-19). **(A)** ADE activity was assessed using a pseudotyped SARS-CoV-2 entry assay across serial plasma dilutions in patients with mild and severe COVID-19. Data are presented as relative luciferase activity normalized to the virus control. Negative control (N.C.) represents pre-pandemic plasma samples. **(B)** ADE activity quantified as area under the curve (AUC) was compared between patients with mild and severe COVID-19. Each dot represents an individual patient; bars indicate mean ± standard error of the mean. Differences between the two groups were analyzed using the Mann–Whitney U test. Sample sizes were as follows: **(A)** mild (n = 40), severe (n = 24), and negative control (n = 5); **(B)** mild (n = 40) and severe (n = 24). All tests were two-sided, and *p*-values < 0.05 were considered statistically significant. ****p < 0.0001.

ADE activity, assessed using pseudotyped SARS-CoV-2 entry into THP-1 monocyte-like cells, was significantly higher in plasma of patients with severe COVID-19 than in those with mild disease (**Figure 2A**). To quantify overall ADE activity across the dilution series, we calculated the AUC for each participant. Severe cases demonstrated substantially higher ADE AUC values than did mild cases (7.47 [IQR, 6.24–10.70] vs. 4.92 [IQR, 4.65–5.15], *p* < 0.0001) (**Figure 2B**). These findings indicate that enhanced FcγR-dependent viral entry is associated with severe COVID-19 and is consistent with ADE-like enhancement of viral uptake in monocyte-like cells.

**Figure 3:**
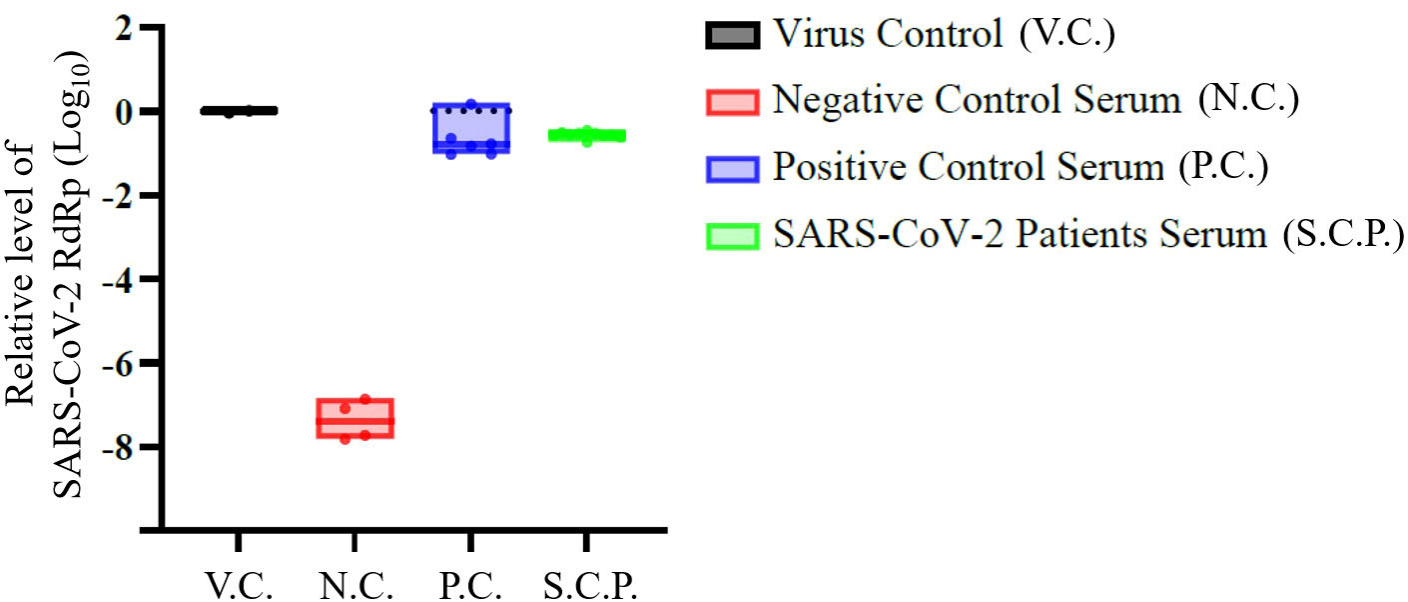
SARS-CoV-2 RdRP levels in THP-1 cells following live-virus infection. Relative levels of SARS-CoV-2 RNA (RdRp) were measured by quantitative PCR and expressed as log_10_-transformed values. Virus control (V.C.) represents infection in the absence of serum. Negative control serum (N.C.) consists of pre-pandemic plasma samples. Positive control serum (P.C.) corresponds to a recombinant SARS-CoV-2 spike monoclonal antibody with reported antibody-dependent enhancement (ADE) activity. SARS-CoV-2 patient serum (S.C.P.) represents plasma obtained from infected individuals. Each dot represents an individual measurement; bars indicate mean ± standard error of the mean. Differences among groups were assessed descriptively. Sample sizes were as follows: V.C. (n = 1), N.C. (n = 2), P.C. (n = 3), and S.C.P. (n = 10). All tests were two-sided, and *p*-values < 0.05 were considered statistically significant.

To distinguish between viral entry and productive replication, we performed live-virus assays using the SARS-CoV-2 BA.5 Omicron variant on the ten serum samples that exhibited the strongest ADE activity. In THP-1 cells, viral RNA (*RdRP*) was detectable in all immune complex-treated groups. The raw Ct values (mean 24.3 ± 0.4) were comparable to those of the virus-only controls, confirming successful viral uptake. However, when normalized to the virus control to assess replication kinetics, no significant increase in relative viral RNA levels (Log_10_ fold-change) was observed at 24 h post-infection (**Figure 3**). This absence of RNA accumulation relative to the inoculum suggests that while patient antibodies facilitate FcγR-mediated entry, the process is restricted to internalization without subsequent propagation, consistent with an abortive infection.

### 3.4 FcγRIIIa-mediated reporter assay (ADCC surrogate) activity

**Figure 4:**
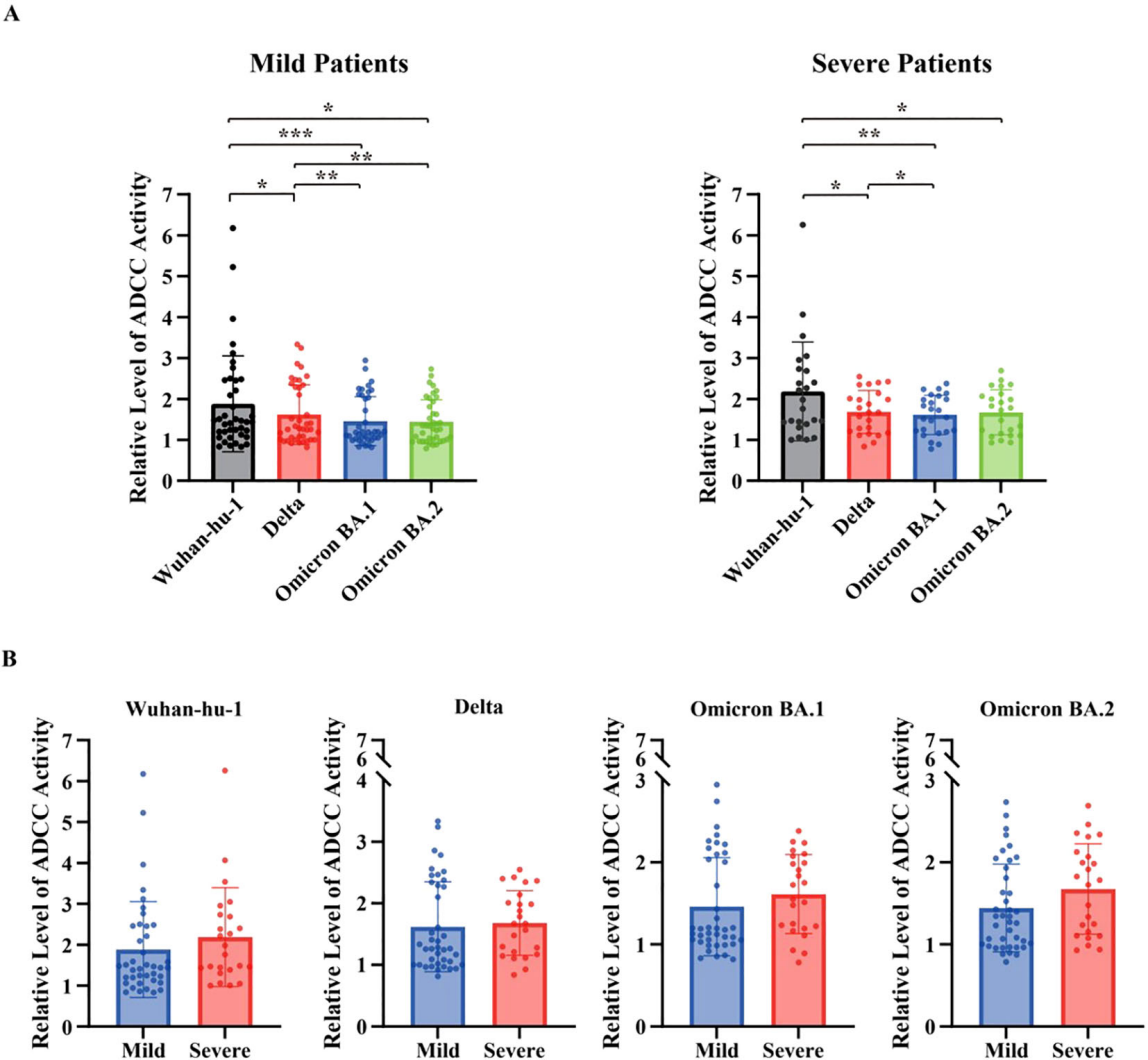
Fc gamma receptor (FcγR)IIIa-mediated reporter activity (ADCC surrogate) against SARS-CoV-2 variants. **(A)** FcγRIIIa-mediated reporter activity (ADCC surrogate) was measured using spike-expressing A549 target cells and Fcγ receptor–expressing Jurkat-Lucia NFAT-CD16 reporter cells across SARS-CoV-2 variants (Wuhan-Hu-1, Delta, Omicron BA.1, and BA.2) in patients with mild and severe COVID-19. Data are presented as relative luminescence. Comparisons among variants within each severity group were performed using one-way repeated measures analysis of variance. **(B)** Comparison of FcγRIIIa-mediated reporter activity between patients with mild and severe COVID-19 for each SARS-CoV-2 variant Each dot represents an individual patient; bars indicate mean ± standard error of the mean. Differences between two groups were analyzed using the Mann–Whitney U test. Sample sizes were as follows: mild (n = 40) and severe (n = 24) patients. All tests were two-sided, and *p*-values < 0.05 were considered statistically significant. *p < 0.05, **p < 0.01, ***p < 0.001, ****p < 0.0001 ADCC, antibody-dependent cellular cytotoxicity; COVID-19, coronavirus disease 2019.

FcγRIIIa-mediated reporter activity, measured as a surrogate for ADCC potential, was assessed using spike-expressing A549 target cells and Fcγ receptor–bearing Jurkat-Lucia reporter cells. As shown in **Figure 4A**, FcγRIIIa-mediated reporter activity responses were highest against the ancestral Wuhan-hu-1 strain and decreased progressively against the Delta and Omicron subvariants in both mild and severe groups. However, pairwise comparisons between the mild and severe disease groups showed no significant differences for Wuhan-hu-1, Delta, or the Omicron subvariants. Against the Wuhan-hu-1 spike, median FcγRIIIa-mediated reporter activity in patients with mild disease was 1.46 (IQR, 1.18–2.45) and 1.87 (IQR, 1.42–2.70) in those with severe disease (*p* = 0.18). Similar patterns were observed for Delta (mild 1.27 [1.01–2.30] vs. severe 1.67 [1.20–2.04], *p* = 0.36), Omicron BA.1 (mild 1.18 [1.05–2.05] vs. severe 1.64 [1.22–2.01], *p* = 0.14), and Omicron BA.2 (mild 1.31 [1.00–1.84] vs. severe 1.76 [1.14–2.08], *p* = 0.10) (**Figure 4B**). Taken together, these findings suggest that FcγRIIIa-mediated reporter activity is primarily influenced by viral antigenic variation rather than clinical disease severity.

### 3.5 FcγRIIa mediated reporter assay (ADCP surrogate) activity

**Figure 5:**
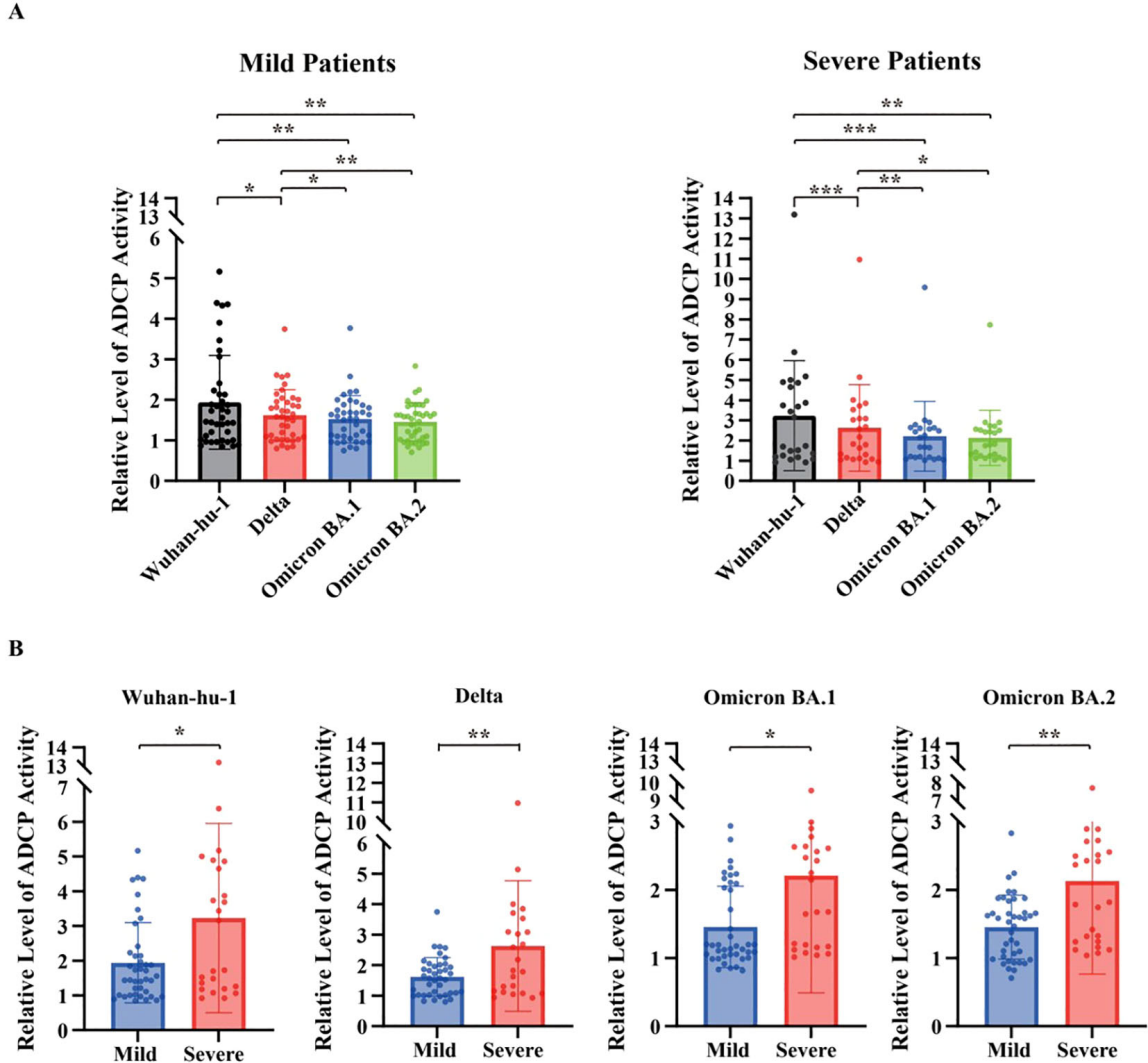
Fc gamma receptor (FcγR)IIa-mediated reporter activity (ADCP surrogate) against SARS-CoV-2 variants. **(A)** FcγRIIa-mediated reporter activity was measured using spike-expressing A549 target cells and Fcγ receptor–expressing Jurkat-Lucia NFAT-CD32 reporter cells across SARS-CoV-2 variants (Wuhan-Hu-1, Delta, Omicron BA.1, and BA.2) in patients with mild and severe COVID-19. Data are presented as relative luminescence. Comparisons among variants within each severity group were performed using one-way repeated measures analysis of variance. **(B)** Comparison of FcγRIIa-mediated reporter activity between patients with mild and severe COVID-19 for each SARS-CoV-2 variant Each dot represents an individual patient; bars indicate mean ± standard error of the mean. Differences between the two groups were analyzed using the Mann–Whitney U test. Sample sizes were as follows: mild (n = 40) and severe (n = 24) patients. All tests were two-sided, and *p*-values < 0.05 were considered statistically significant. *p < 0.05, **p < 0.01, ***p < 0.001 COVID-19, coronavirus disease 2019; ADCP, antibody-dependent cellular phagocytosis.

FcγRIIa-mediated reporter activity, measured as a surrogate for ADCP potential, was evaluated using the same target cell system with Jurkat-Lucia reporter cells as effector cells. As shown in **Figure 5A**, FcγRIIa-mediated reporter activity was also progressively lower in the Delta and Omicron subvariants than in the ancestral Wuhan-hu-1 strain in both mild and severe disease groups. Nonetheless, absolute FcγRIIa-mediated reporter activity levels were generally higher in patients with severe disease than in those with mild disease across the tested variants. For example, against the Wuhan-hu-1 spike, the median FcγRIIa-mediated reporter activity was 1.51 (IQR 1.02–2.16) in patients with mild disease versus 2.44 (IQR 1.24–4.70) in those with severe disease (*p* = 0.066), although this difference did not reach statistical significance. In contrast, significantly higher FcγRIIa-mediated reporter activity was observed for Delta (mild 1.58 [1.07–1.97] vs. severe 2.00 [1.14–3.20], *p* = 0.038), Omicron BA.1 (mild 1.54 [1.09–1.82] vs. severe 1.91 [1.17–2.62], *p* = 0.019), and Omicron BA.2 (mild 1.56 [1.01–1.71] vs. severe 1.80 [1.24–2.52], *p* = 0.008) (**Figure 5B**). These results indicate that FcγRIIa-mediated reporter activity was elevated in severe cases, potentially reflecting enhanced FcγR-mediated immune activation associated with inflammatory landscape of severeCOVID-19.

### 3.6 Univariate and multivariate logistic regression analyses of Fc-mediated antibody functions

Univariate logistic regression showed that higher ADE activity was strongly associated with severe disease (OR 8.93, 95% CI 3.17–38.67, *p* < 0.001). FcγRIIIa-mediated reporter activity (ADCC surrogate) was not associated with severe COVID-19 (OR 1.24, 95% CI 0.80–1.94, *p* = 0.33), whereas FcγRIIa-mediated reporter activity (ADCP surrogate) demonstrated a significant association with severe COVID-19 (OR 1.52, 95% CI 1.10–2.24, *p* = 0.022) (**Table 2**).

**Table 2:** Univariate and multivariable logistic regression analyses of Fc-mediated antibody functions associated with severe coronavirus disease 2019 (COVID-19).

Fc-mediated antibody functions	Univariate OR (95% CI)	*p*-value	Multivariate OR (95% CI)*	*p*-value
ADE	8.93 (3.17–38.67)	< 0.001	1.10 × 10^5^ (6.13 × 10¹–4.43 × 10¹^0^)	0.022
FcγRIIIa mediated reporter assay (ADCC surrogate)	1.24 (0.80–1.94)	0.331	1.71 (0.30–10.60)	0.545
FcγRIIa mediated reporter assay (ADCP surrogate)	1.52 (1.10–2.24)	0.022	2.37 (0.72–10.19)	0.192

*Multivariate models adjusted for age, sex, variant period, and comorbidity status.

ADE, antibody-dependent enhancement; ADCC, antibody-dependent cellular cytotoxicity; ADCP, antibody-dependent cellular phagocytosis; OR, odds ratio; CI, confidence interval; FcγR, Fc gamma receptor.

In multivariable logistic regression adjusting for age, sex, variant period, and comorbidity status, higher ADE activity remained independently associated with severe COVID-19 (adjusted OR 1.10 × 10^5^, 95% CI 6.13 × 10¹ to 4.43 × 10¹^0^, *p* = 0.022), although the CI was notably wide due to the sample size and distribution. In the same multivariable model, neither FcγRIIIa-mediated (adjusted OR 1.71, 95% CI 0.30–10.60, *p* = 0.54), nor FcγRIIa-mediated reporter activity (adjusted OR 2.37, 95% CI 0.72–10.19, *p* = 0.19) showed a significant independent association with disease severity (**Table 2**).

When ADE and FcγRIIa-mediated reporter activities were included together in the multivariable model, ADE retained its significant association (adjusted OR 1.29 × 10^5^, 95% CI 5.44 × 10¹ to 9.93 × 10¹^0^, *p* = 0.024), while FcγRIIa-mediated reporter activity did not (adjusted OR 1.38, 95% CI 0.12–16.41, *p* = 0.79) (data not shown). These findings indicate that ADE showed the strongest independent association with severe disease. The univariate association observed for FcγRIIa-mediated reporter activity (ADCP surrogate) likely reflects overlapping Fc-mediated immune signatures dominated by the ADE response rather than an independent pathogenic contribution.

### 3.7 Comparison of Fc-mediated antibody responses pre- Omicron and post-Omicron groups

Plasma IgG binding to the SARS-CoV-2 spike S1 domain was highest against the ancestral Wuhan strain in both groups (mild and severe disease) compared with that against other variants. However, when responses were analyzed within each variant, no significant differences were observed between the pre- Omicron and post- Omicron groups (**Supplementary Figure 1**).

For FcγRIIIa-mediated reporter activity, no differences were observed among variants within the pre- Omicron group, whereas in the post- Omicron group, activity was highest against the ancestral Wuhan strain. Nevertheless, when directly compared across individual variants, no significant differences were noted between the pre and post Omicron groups (**Supplementary Figure 2**). Similarly, FcγRIIa-mediated reporter activity was highest against the ancestral Wuhan strain in both groups, mirroring the pattern observed for spike S1 binding. Although one outlier was noted, stratification by individual variants again revealed no significant or reproducible differences between the pre-Omicron and post- Omicron groups (**Supplementary Figure 3**).

Taken together, these results suggest that reduced Fc-mediated effector activity against Omicron subvariants is primarily related to changes in spike protein antigenicity than to differences in prior exposure or exposure timing.

### 3.8 Association between ADE activity and systemic inflammatory markers and circulating cytokines

To investigate whether ADE activity is associated with inflammatory responses in COVID-19, we analyzed both systemic inflammatory markers and circulating cytokines in patient plasma samples. CRP and NLR levels were significantly higher in patients with severe COVID-19 than in those with mild disease. Furthermore, ADE activity showed significant positive correlations with both CRP and NLR, indicating that higher ADE activity is associated with increased systemic inflammation (**Supplementary Figure 4**). We next measured circulating pro-inflammatory cytokines, including IL-6 and TNF-α. Both cytokines were significantly elevated in patients with severe disease than in those with mild disease. Importantly, ADE activity was positively correlated with circulating IL-6 and TNF-α levels (**Supplementary Figure 5**). These findings indicate that higher ADE activity is associated with systemic inflammatory markers and circulating cytokine levels, suggesting a link between FcγR-mediated antibody responses and inflammatory status in COVID-19.

## 4 Discussion

Fc-mediated antibody effector functions represent a critical interface between antiviral immunity and immunopathology in COVID-19. In our cohort, ADE activity showed a clear and independent association with severe disease. In contrast FcγRIIa-mediated reporter activity (a surrogate for ADCP)—although higher in severe cases in crude comparisons—lost statistical significance after adjusting for confounding factors. FcγRIIIa-mediated reporter activity (a surrogate for ADCC) showed no association with disease severity in any analytical approach. These findings support a model in which excessive FcγR engagement may serve as a prominent functional marker of the dysregulated immune response in severe COVID-19 (25, 26).

Pseudovirus-based ADE assays demonstrated stronger FcγR-dependent viral entry in plasma from patients with severe disease than in patients with mild disease, suggesting enhanced antibody–FcγR engagement. However, live-virus experiments using Omicron variants did not show increases in viral RNA replication, consistent with an entry-type rather than replication-type ADE mechanism. This finding aligns with the model proposed by Junqueira et al. (27) in which SARS-CoV-2 enters FcγR-expressing monocytes via an immune complex without completing its replication cycle, potentially leading to inflammasome activation and pro-inflammatory cytokine release. Collectively, our results support the interpretation that FcγR-dependent viral entry may be a potential functional marker of immunopathology, likely through altered myeloid activation rather than productive viral replication. This activation is critical, as the engagement of FcγRs on myeloid cells is known to initiate potent downstream intracellular signaling cascades. Specifically, pathways such as NF-κB and MAP kinase are typically triggered, leading to the rapid transcription and release of a broad spectrum of pro-inflammatory cytokines and chemokines, including IL-6, TNF-α, and IL-1β (28). While our study did not directly measure cytokine production or intracellular signaling events, we observed that ADE activity was positively associated with both systemic inflammatory markers (CRP and NLR) and circulating cytokines (IL-6 and TNF-α), supporting a link between Fc-mediated functionality and the hyperinflammatory state of severe COVID-19. However, these associations were largely driven by differences between severity groups, and cytokine levels showed overlap between strata, suggesting that these relationships may reflect broader clinical phenotypes rather than a direct, individual-level causal link.

Both patient groups (mild and severe COVID-19) exhibited markedly reduced IgG binding to all tested variants than to the wild-type strain, reflecting the substantial impact of spike protein mutations on antigenicity. Binding responses did not differ across severity groups, consistent with the lack of FcγRIIIa-mediated reporter activity differences between clinical phenotypes. FcγRIIIa-mediated reporter activity remained unaffected by disease severity in both descriptive and regression analyses, suggesting that FcγRIIIa-mediated reporter is largely driven by antigenic variation rather than clinical outcome. In contrast, FcγRIIa-mediated reporter activity was higher in patients with severe disease in crude between-group comparisons and univariate logistic regression, but lost significance after multivariable adjustment. When ADE and FcγRIIa-mediated reporter activity were modeled together, only ADE retained an independent association with severe disease. These data suggest that FcγRIIa-mediated reporter activity may reflects confounding or overlapping Fc-mediated immune programs dominated by the much stronger ADE response, rather than an independent effect on disease severity—a phenomenon consistent with prior reports that phagocytic and other Fc-effector signatures often lose significance once broader immune activation and antibody subclass differences are considered (29, 30).

When Fc-effector responses were compared between patients diagnosed before and after the emergence of the Omicron variant, no significant differences were observed in FcγRIIIa-mediated reporter or FcγRIIa-mediated reporter activities within matched variant groups. This finding suggests that temporal factors—such as vaccination, hybrid immunity, or population-level exposure— did not substantially modify Fc-effector quality once antigenic context was considered. Therefore, the observed decline in Fc-effector activity against Omicron sublineages is more likely related to structural remodeling of the spike protein rather than immunologic fatigue or waning immunity. Consistent with recent systems-serology analyses, Fc-functionality appears to be primarily determined by epitope accessibility and FcγR-binding architecture rather than by infection timing or vaccine dose intervals (31, 32). These results emphasize that antigenic evolution, more than chronological or immunologic factors, may represent a key factor shaping Fc-effector reconfiguration in the Omicron era.

Qualitative features of the IgG Fc domain substantially influence downstream inflammatory responses during SARS-CoV-2 infection. Early anti–SARS-CoV-2 IgG with low fucosylation exhibits enhanced binding to FcγRIIIa and FcγRIIa on monocytes and macrophages, driving exaggerated cytokine production and alveolar inflammation—a pattern repeatedly associated with severe disease (33, 34). Thus, Fc-dependent effector mechanisms are not exclusively protective but may potentiate inflammatory cascades under specific glycosylation and FcγR-engagement conditions. Fc-effector pathway integrity also appears essential for effective antiviral activity, as Fc-compromised monoclonal antibodies show diminished *in vivo* protection despite preserved neutralizing activity (35). These observations reinforce the concept that Fc-mediated responses operate within interconnected immunologic networks rather than in isolation. In this context, our finding that FcγRIIa-mediated reporter activity lost significance after variable adjustment—or when modeled alongside ADE—suggests that FcγRIIa-mediated reporter activity (as a surrogate for ADCP) more likely reflects upstream Fc-driven activation patterns dominated by ADE rather than an independent pathogenic mechanism. Although ADE retained a significant association with severe disease, the wide confidence interval around its effect estimate likely reflects both the limited sample size and the inherently skewed distribution of ADE responses. Thus, while the direction of the association was consistent, the imprecision of the estimate indicates that the absolute magnitude of the risk should be interpreted cautiously.

As of this writing (October 2025), SARS-CoV-2 continues to circulate in South Korea with a recent increase in hospitalizations and sustained community transmission predominantly attributed to Omicron sublineages NB.1.8.1 and LP.8.1 (36). Despite a substantial decline in overall COVID-19 mortality compared with that observed in previous pandemic waves, infection remains a source of significant morbidity, especially among immunocompromised individuals and other high-risk groups (37). Thus, prevention remains a priority, and while various vaccines have been developed with demonstrated effectiveness, the challenges posed by ongoing viral evolution persist. Recent studies have shown that COVID-19 vaccination enhances Fc-mediated responses, increasing antibody affinity for FcγRs on NK cells and macrophages (31, 38, 39). However, the ultimate impact of these enhanced Fc functions on disease outcomes is complex, as they can promote both protective antiviral activity and, in certain contexts, maladaptive inflammation that contributes to tissue damage. Therefore, continued investigation into Fc-mediated immunity remains essential for informing strategies to protect vulnerable individuals and mitigate the impact of emerging variants.

Compared with previous studies, our work offers several distinct strengths. First, most previous reports examined individual FcγR-mediated activities in isolation, whereas we simultaneously quantified ADE, FcγRIIIa- and FcγRIIa-mediated reporter activities in a prospectively enrolled clinical cohort, enabling an integrated assessment of Fc-effector profiles across the full spectrum of COVID-19 severity. Second, the study population was drawn from two independent hospitals and included patients diagnosed both before and after the emergence of the Omicron variant, enhancing the generalizability and clinical relevance of our findings. Third, by extending the analysis to multiple viral variants—including Omicron and its sublineages—we provide additional insight into how antigenic evolution may influence Fc-mediated immunity, an area that has been less frequently evaluated in previous Fc-function studies. Nonetheless, this study has some limitations. The modest sample size limited the granularity of subgroup analyses and may have reduced statistical power, particularly in variant-stratified and multivariable models. As such, the associations identified here should be interpreted with caution, and validation in larger, independently recruited cohorts will be important. Additionally, we did not directly assess intracellular signaling events downstream of FcγR engagement. It is important to note that FcγRIIIa- and FcγRIIa-mediated reporter assays used in this study provide a measure of receptor activation potential rather than direct quantification of cellular cytotoxicity or phagocytosis. Our experiments were designed to characterize functional antibody activities rather than delineate molecular pathways; therefore, the findings should be considered in the context of previously characterized FcγR-dependent inflammatory mechanisms. Future studies incorporating Fc glycosylation profiling and FcγR polymorphism analyses—both of which modulate Fc-effector activity—will be valuable for clarifying the upstream determinants of ADE- and ADCP-related functional responses (40). Information on prior SARS-CoV-2 vaccination or previous infection was not consistently available for all participants. As prior antigen exposure can shape antibody repertoires and Fc-mediated functional responses, we cannot exclude that individual immune history might have influenced the observed results. Moreover, longitudinal sampling and integration of cellular immune profiling will be critical to distinguish protective from pathogenic Fc-mediated effects throughout infection. Finally, antibody-dependent complement deposition (ADCD) was not assessed in this study because complement-dependent assays require freshly collected, non–heat-inactivated samples and standardized complement sources, which were not compatible with the plasma-based FcγR assays used here.

In summary, our findings highlight the differential contributions of Fc-mediated antibody functions to COVID-19 severity. ADE activity emerged as the prominent functional marker independently associated with severe disease, supporting a potential role for excessive FcγR engagement in driving pathogenic myeloid activation. While FcγRIIa-mediated reporter activity (a surrogate for ADCP) was higher among severe cases than among mild cases in unadjusted analyses, it lost significance after multivariable adjustment or when modeled alongside ADE. This suggests that FcγRIIa activation reflects broader, overlapping Fc-driven immune signatures rather than an independent determinant of severity. In contrast, FcγRIIIa-mediated reporter activity (a surrogate for ADCC) declined with antigenic evolution but showed no meaningful association with clinical outcomes. Together, these observations underscore the complexity of Fc-effector immunity in SARS-CoV-2 infection and emphasize the need to precisely modulate Fc–FcγR interactions to mitigate maladaptive inflammation in severe cases. Further mechanistic investigation will be essential to refine the design of next-generation antibody-based therapeutics that balance protective immunity and immunopathological risks.

## Data Availability

The original contributions presented in the study are included in the article/**Supplementary Material**. Further inquiries can be directed to the corresponding author.

## Supplementary material

Supplementary Figure 1 Plasma IgG binding before and after Omicron predominance. **(A)** Plasma IgG binding to the SARS-CoV-2 spike S1 protein across variants (Wuhan-Hu-1, B.1.1.529, BA.4/BA.5/BA.5.2, and XBB1.5) in patients diagnosed before and after Omicron predominance. Data are presented as relative optical density (OD) at 450 nm. Comparisons among variants within each group were performed using one-way repeated measures ANOVA. **(B)** Comparison of plasma IgG binding between the Before Omicron and After Omicron groups for each SARS-CoV-2 variant. Each dot represents an individual patient; bars indicate mean ± SEM. Differences between the two groups were analyzed using the Mann–Whitney U test. Sample sizes were as follows: Before Omicron (n = 21) and After Omicron (n = 43). All tests were two-sided, and p-values < 0.05 were considered statistically significant.

Supplementary Figure 2 ADCC activity before and after Omicron predominance. **(A)** ADCC activity was measured using spike-expressing A549 target cells and Fcγ receptor–expressing Jurkat-Lucia NFAT-CD16 reporter cells across SARS-CoV-2 variants (Wuhan-Hu-1, Delta, Omicron BA.1 and BA.2) in patients diagnosed before and after Omicron predominance. Data are presented as relative luminescence. Comparisons among variants within each group were performed using one-way repeated measures ANOVA. **(B)** Comparison of ADCC activity between the Before Omicron and After Omicron groups for each SARS-CoV-2 variant. Each dot represents an individual patient; bars indicate mean ± SEM. Differences between the two groups were analyzed using the Mann–Whitney U test. Sample sizes were as follows: Before Omicron (n = 21) and After Omicron (n = 43). All tests were two-sided, and p-values < 0.05 were considered statistically significant. **p* < 0.05, ***p* < 0.01, ****p* < 0.001.

Supplementary Figure 3 ADCP activity before and after Omicron predominance. **(A)** Antibody-dependent cellular phagocytosis (ADCP) activity was measured using spike-expressing A549 target cells and Fcγ receptor–expressing Jurkat-Lucia NFAT-CD32 reporter cells across SARSCoV-2 variants (Wuhan-Hu-1, Delta, Omicron BA.1 and BA.2) in patients diagnosed before and after Omicron predominance. Data are presented as relative luminescence. Comparisons among variants within each group were performed using one-way repeated measures ANOVA. **(B)** Comparison of ADCP activity between the Omicron and After Omicron groups for each SARSCoV-2 variant. Each dot represents an individual patient; bars indicate mean ± SEM. Differences between the two groups were analyzed using the Mann–Whitney U test. Sample sizes were as follows: Before Omicron (n = 21) and After Omicron (n = 43). All tests were two-sided, and p-values < 0.05 were considered statistically significant. **p* < 0.05, ***p* < 0.01, ****p* < 0.001.

Supplementary Figure 4 Association of ADE activity with systemic inflammatory markers. **(A–B)** Plasma C-reactive protein (CRP) levels and neutrophil-to-lymphocyte ratio (NLR) in patients with mild and severe COVID-19. Each dot represents an individual patient; bars indicate mean ± SEM. Differences between the two groups were analyzed using the Mann–Whitney U test. Sample sizes were as follows: mild (n = 30) and severe (n = 22). **(C–D)** Correlations between antibody-dependent enhancement (ADE) activity (AUC) and CRP and NLR levels. Each dot represents an individual patient. Correlations were evaluated using Spearman’s rank correlation coefficient. The total sample size was n = 52 (mild, n = 30; severe, n = 22). All tests were two-sided, and p-values < 0.05 were considered statistically significant. **p* < 0.05, ***p* < 0.01, ****p* < 0.001.

Supplementary Figure 5 Association of ADE activity with circulating cytokines. **(A–B)** Interleukin-6 (IL-6) and tumor necrosis factor-alpha (TNF-α) levels in patients with mild and severe COVID-19. Each dot represents an individual patient; bars indicate mean ± SEM. Differences between the two groups were analyzed using the Mann–Whitney U test. Sample sizes were as follows: mild (n = 24) and severe (n = 40). **(C–D)** Correlations between antibody-dependent enhancement (ADE) activity (AUC) and IL-6 and TNF-α levels. Each dot represents an individual patient. Correlations were evaluated using Spearman’s rank correlation coefficient. The total sample size was n = 64 (mild, n = 24; severe, n = 40). All tests were two-sided, and p-values < 0.05 were considered statistically significant. **p* < 0.05, ***p* < 0.01, ****p* < 0.001.
