# Supplementary material for: Fc-mediated antibody functions are associated with disease severity in COVID-19
Source: Front Immunol. 2026 May 1;17:1797975. doi: 10.3389/fimmu.2026.1797975 (PMC13176313; doi:10.3389/fimmu.2026.1797975)

Supplemental figure 1

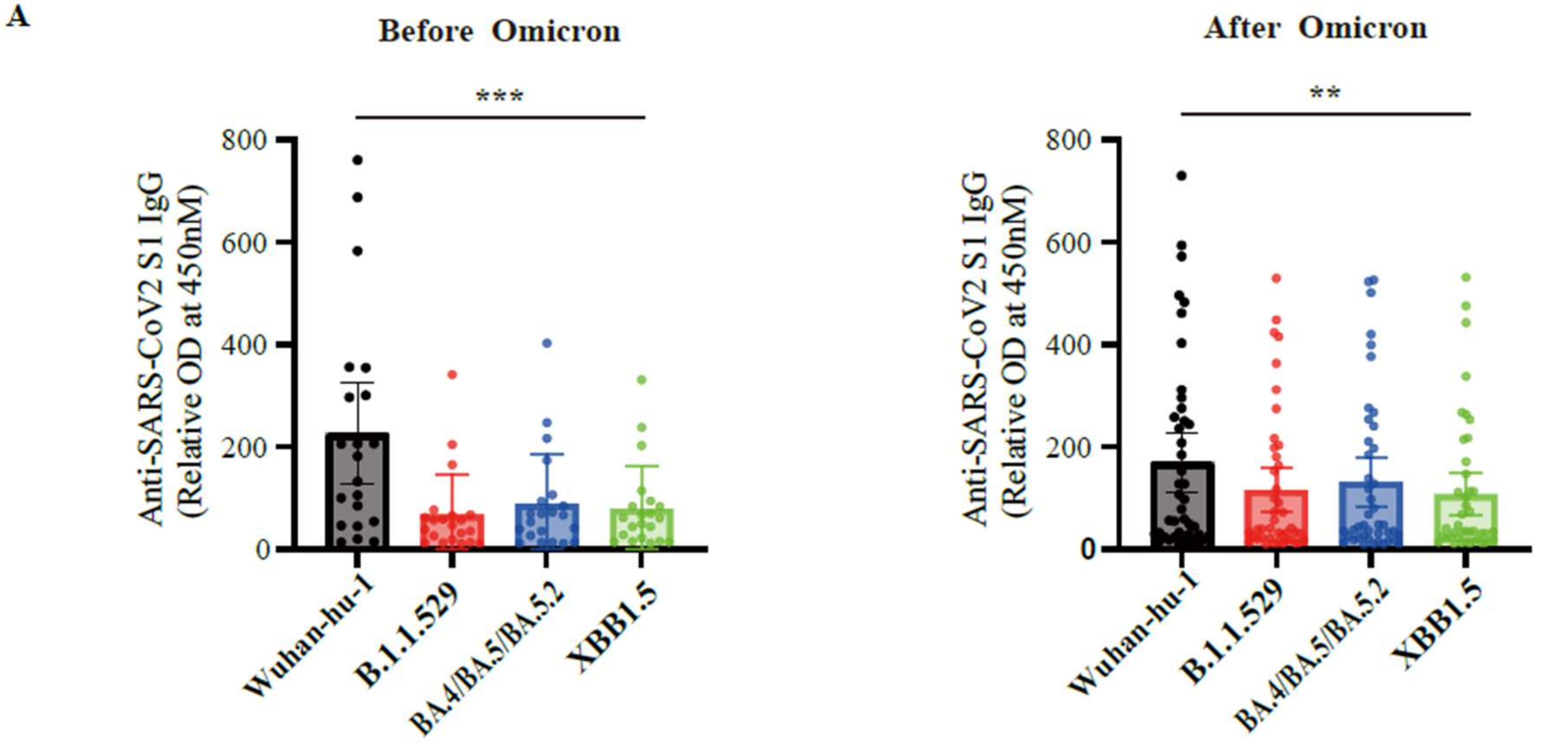

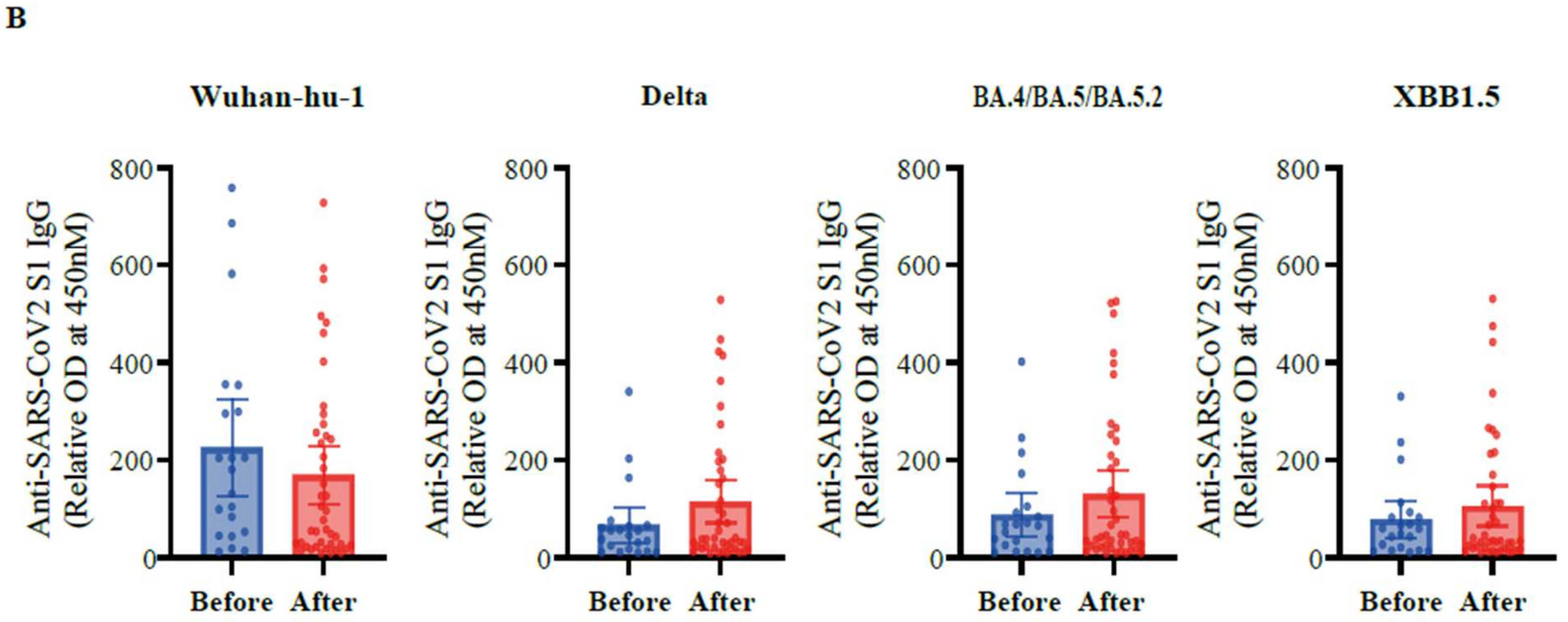

Supplemental figure 2

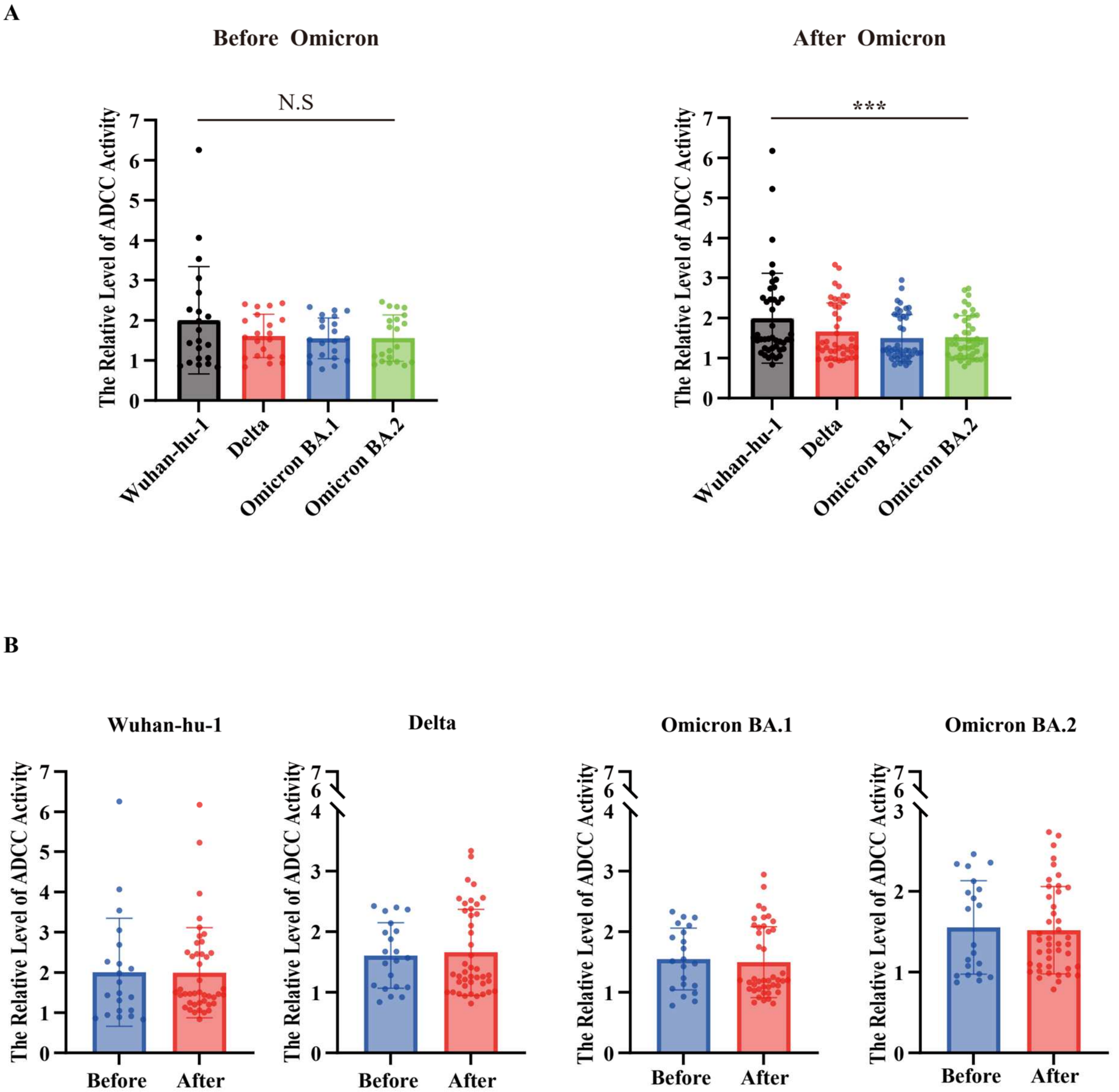

Supplemental figure 3

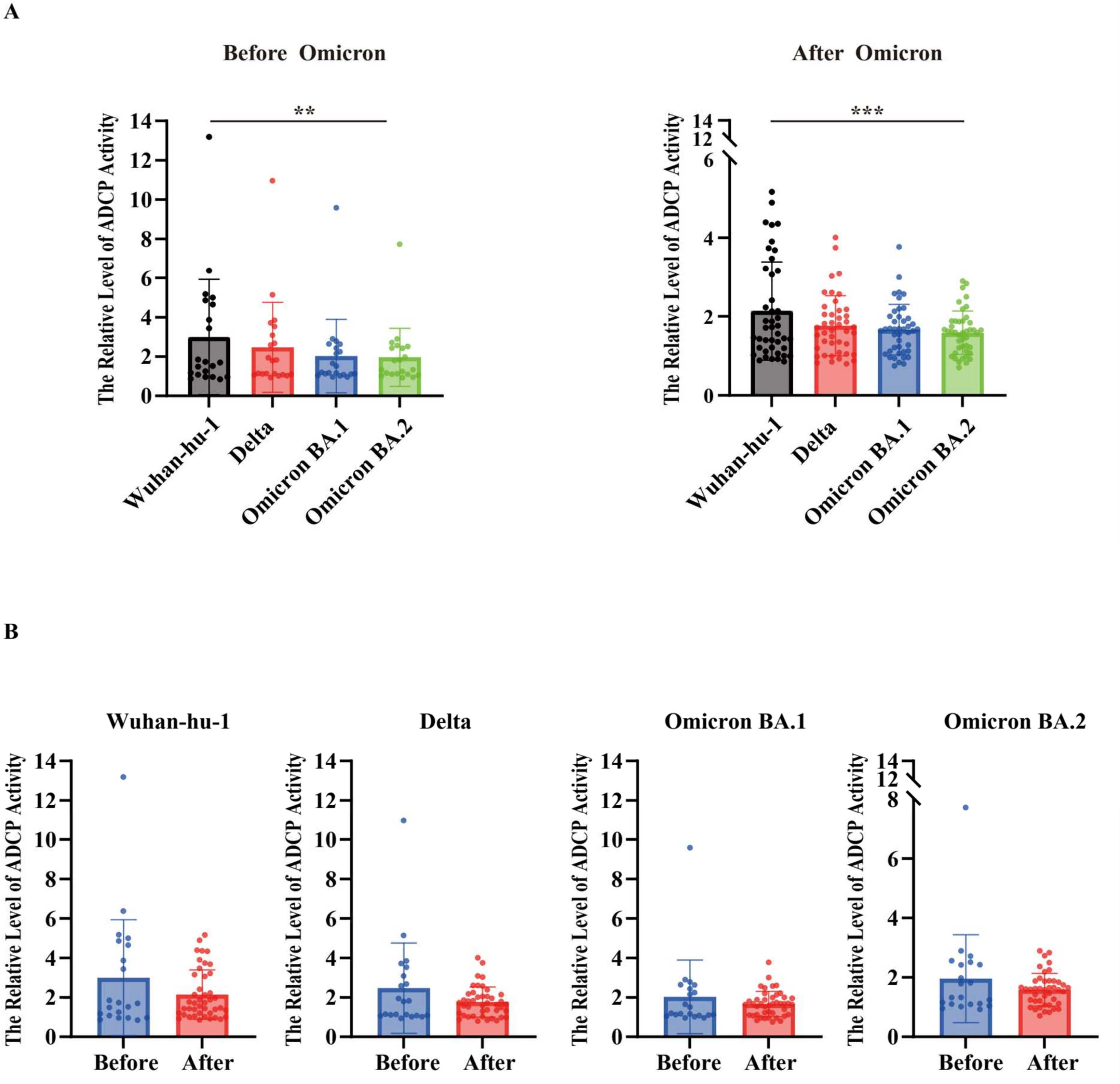

**Supple-
mental
figure 4**

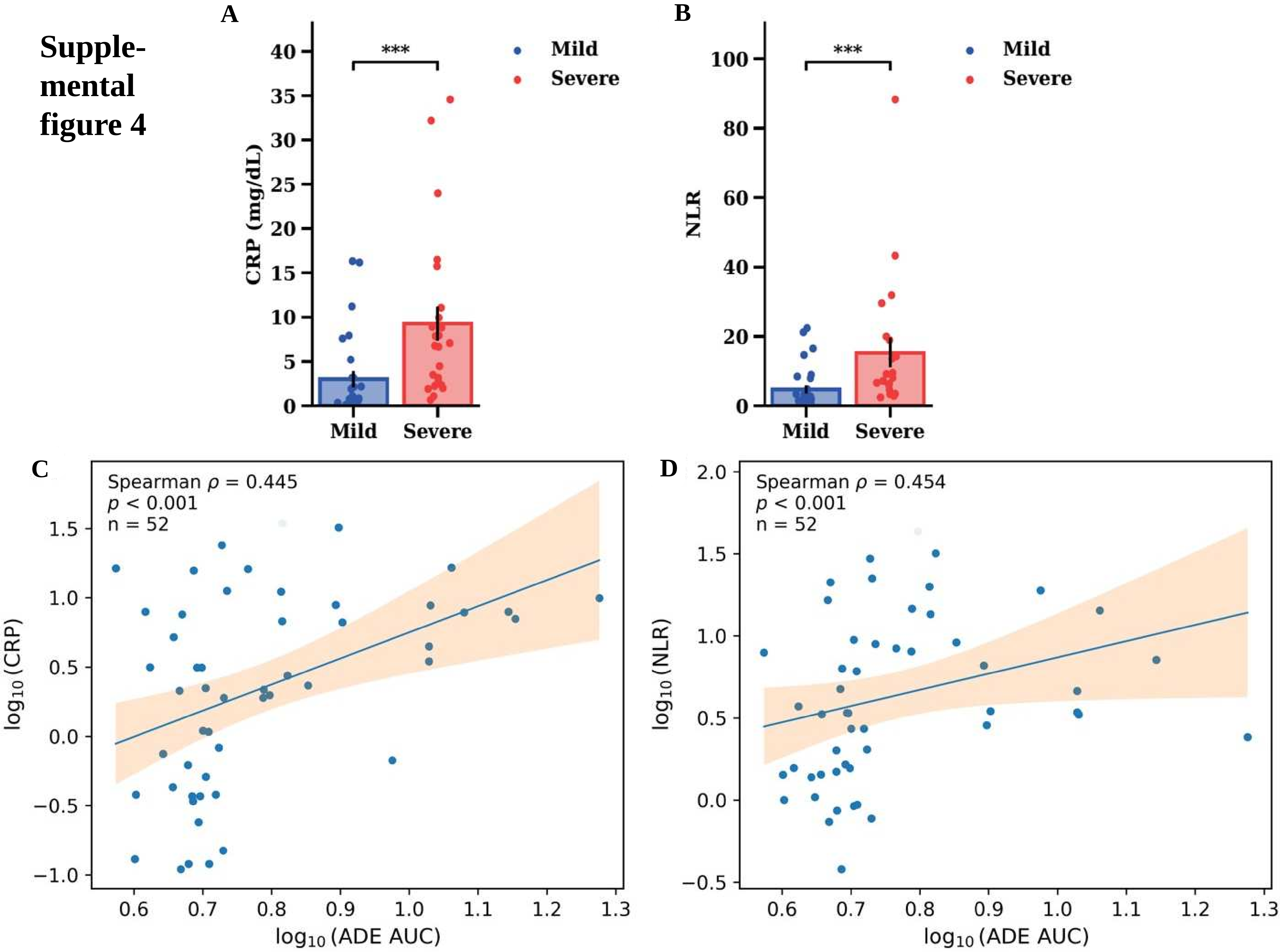

Supple-mental figure 5

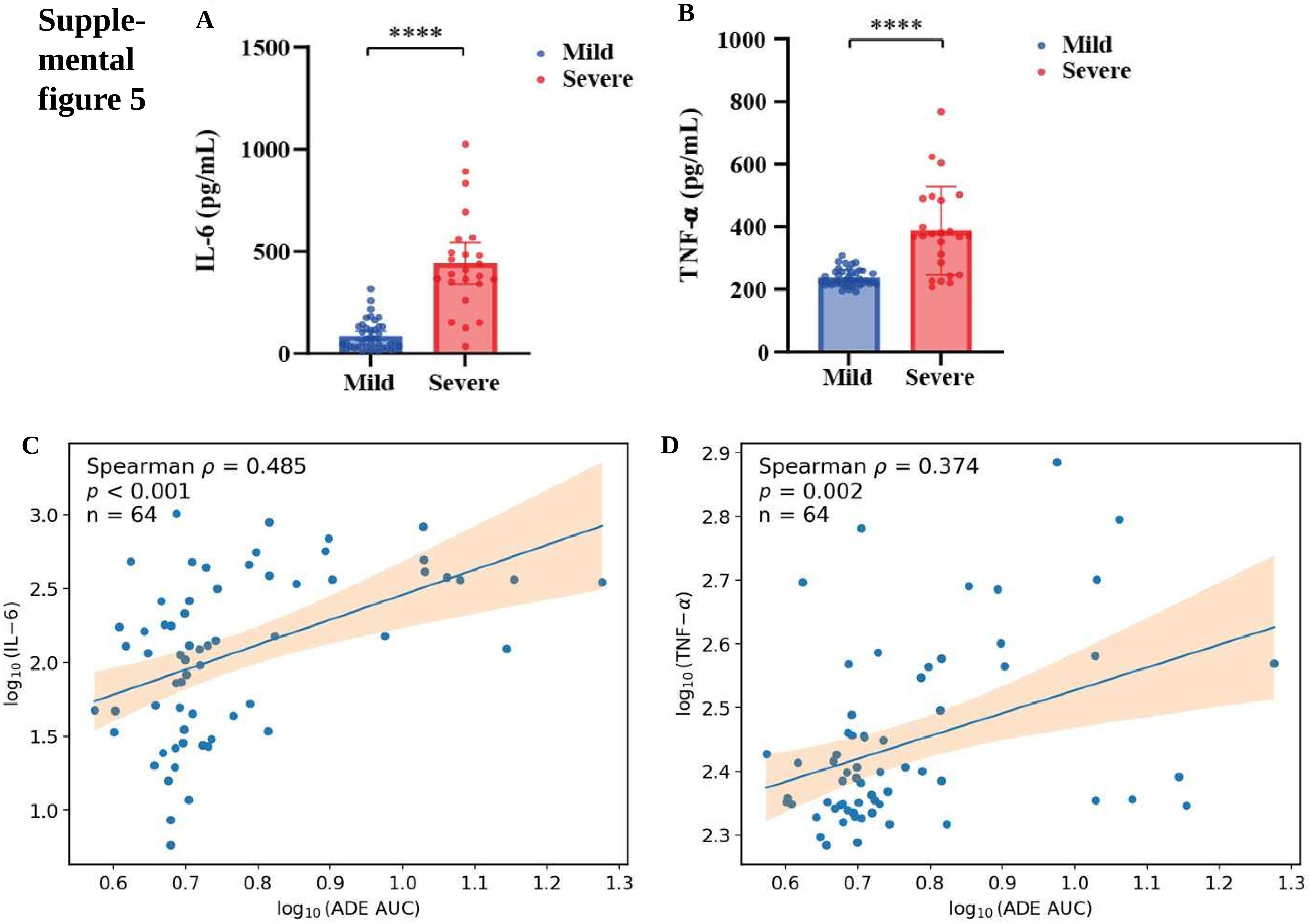

Supplement: Supplementary Figure 1 — Plasma IgG binding before and after Omicron predominance. (A) Plasma IgG binding to the SARS-CoV-2 spike S1 protein across variants (Wuhan-Hu-1, B.1.1.529, BA.4/BA.5/BA.5.2, and XBB1.5) in patients diagnosed before and after Omicron predominance. Data are presented as relative optical density (OD) at 450 nm. Comparisons among variants within each group were performed using one-way repeated measures ANOVA. (B) Comparison of plasma IgG binding between the Before Omicron and After Omicron groups for each SARS-CoV-2 variant. Each dot represents an individual patient; bars indicate mean ± SEM. Differences between the two groups were analyzed using the Mann–Whitney U test. Sample sizes were as follows: Before Omicron (n = 21) and After Omicron (n = 43). All tests were two-sided, and p-values < 0.05 were considered statistically significant. [file DataSheet1.pdf]
